# Unseen and Unprepared: Evaluating Undergraduate Training for Suicide Risk Assessment in UK Medical Schools

**DOI:** 10.1192/bjo.2026.11282

**Published:** 2026-06-30

**Authors:** Nathan Roy

**Affiliations:** University of Bristol Medical School, Bristol, United Kingdom

## Abstract

**Aims:**

Suicide remains a leading cause of death among young adults in the UK, and junior doctors are frequently involved in the early assessment of suicide risk across clinical settings. This study aimed to examine the extent to which undergraduate medical education in the UK prepares students for suicide risk assessment at graduation, and to identify gaps between expected competencies and reported preparedness.

**Methods:**

A structured literature and policy review was conducted using UK-based sources published between 2010 and 2025. Databases included PubMed and Google Scholar, alongside national guidance from the General Medical Council and the Royal College of Psychiatrists. Eligible sources addressed undergraduate medical education, suicide prevention training, curriculum structure, assessment methods, or student preparedness. Evidence was synthesised thematically, focusing on curriculum coverage, skills-based teaching, supervised clinical exposure, assessment, and reported confidence in suicide risk assessment. This work did not involve patients or service users and did not require ethical approval.

**Results:**

The review identified substantial variability in undergraduate training for suicide risk assessment across UK medical schools. While national outcomes frameworks emphasise competence in mental health assessment, suicide risk training is often deliveredinconsistently, with limited protected curriculum time. Common gaps included insufficient skills-based practice, limited supervised exposure to suicide risk assessments during clinical placements, and minimal formal assessment or structured feedback. Across studies, graduating students frequently reported low confidence in conducting suicide risk assessments independently, despite recognising its importance in clinical practice. Where structured, longitudinal teaching was embedded, students demonstrated greater confidence and perceived preparedness.

**Conclusion:**

Undergraduate training for suicide risk assessment in UK medical schools remains variable and frequently insufficient. Greater alignment between national competency expectations and local curriculum delivery is required. Embedding structured, assessed, and longitudinal suicide risk education, supported by supervised clinical exposure and feedback, may improve graduate preparedness, clinician confidence, and patient safety. Strengthening undergraduate education in this area represents a feasible and high-impact opportunity to support earlier identification of suicide risk and safer clinical care.

